# Non-muscle myosin II in disease: mechanisms and therapeutic opportunities

**DOI:** 10.1242/dmm.022103

**Published:** 2015-12-01

**Authors:** Karen A. Newell-Litwa, Rick Horwitz, Marcelo L. Lamers

**Affiliations:** 1 Department of Cell Biology, University of Virginia, Charlottesville, VA 22908, USA; 2 Department of Morphological Sciences, Institute of Basic Health Science, Federal University of Rio Grande do Sul, Porto Alegre, Rio Grande do Sul 90610-010, Brazil

**Keywords:** Migration, Myosin, Stem cell, Synapse, NMII

## Abstract

The actin motor protein non-muscle myosin II (NMII) acts as a master regulator of cell morphology, with a role in several essential cellular processes, including cell migration and post-synaptic dendritic spine plasticity in neurons. NMII also generates forces that alter biochemical signaling, by driving changes in interactions between actin-associated proteins that can ultimately regulate gene transcription. In addition to its roles in normal cellular physiology, NMII has recently emerged as a critical regulator of diverse, genetically complex diseases, including neuronal disorders, cancers and vascular disease. In the context of these disorders, NMII regulatory pathways can be directly mutated or indirectly altered by disease-causing mutations. NMII regulatory pathway genes are also increasingly found in disease-associated copy-number variants, particularly in neuronal disorders such as autism and schizophrenia. Furthermore, manipulation of NMII-mediated contractility regulates stem cell pluripotency and differentiation, thus highlighting the key role of NMII-based pharmaceuticals in the clinical success of stem cell therapies. In this Review, we discuss the emerging role of NMII activity and its regulation by kinases and microRNAs in the pathogenesis and prognosis of a diverse range of diseases, including neuronal disorders, cancer and vascular disease. We also address promising clinical applications and limitations of NMII-based inhibitors in the treatment of these diseases and the development of stem-cell-based therapies.

## Abstract (precis)

**Summary:** Non-muscle myosin II (NMII) contributes to diverse diseases, including neuronal disorders, cancer and vascular disease. We address the role of NMII in disease pathology and highlight promising clinical applications of NMII inhibitors for disease and stem-cell-based therapies.

## Introduction

**Fig. 1.**
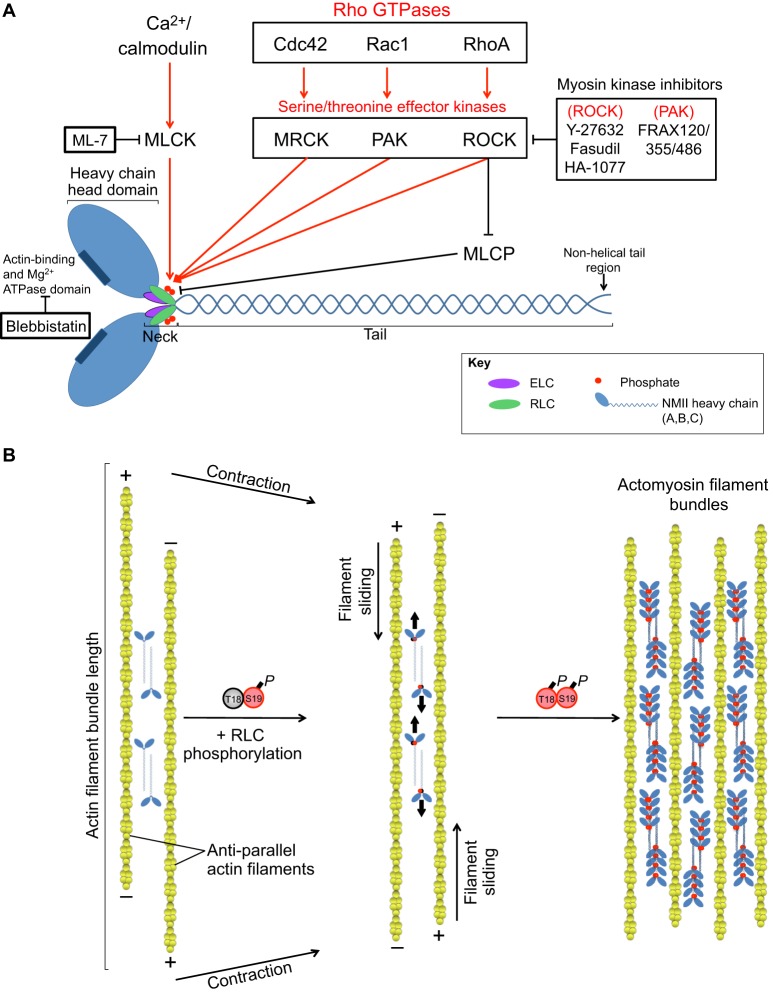
**The structure of** **NMII and its regulation by serine/threonine kinases.** (A) NMII consists of a heavy chain, which includes a globular head domain that binds both actin and ATP; a neck region, which binds both the essential and regulatory light chains (ELC and RLC, respectively); and a tail region, which homodimerizes in a helical fashion, as well as a non-helical tail region that directs the subcellular localization of the NMII isoform. Serine/threonine kinases regulate NMII activity by phosphorylation of the myosin RLC on residues Thr18 and Ser19. These kinases function downstream of small Rho GTPases, as well as downstream of Ca^2+^/calmodulin signaling pathways. This figure also indicates pharmacological inhibitors of myosin regulatory kinases that can be used to modulate NMII activity. (B) NMII filaments associate with each other in an anti-parallel fashion, allowing them to crosslink and slide actin filaments past each other. RLC Ser19 phosphorylation increases NMII ATPase activity, leading to contraction of actin filament bundles, and phosphorylation of both Ser19 and Thr18 increases NMII ATPase activity, driving the association of multiple actin filaments into actomyosin filament bundles, often referred to as stress fibers. MLCK, myosin light chain kinase; MRCK, myotonic dystrophy kinase-related Cdc42-binding kinase; PAK, p21-associated kinase; ROCK, RhoA-associated kinase; MLCP, myosin light chain phosphatase.

Non-muscle myosin II (NMII) is a major contributor to cellular organization, polarity and regulation, with altered NMII activity contributing to numerous disease pathologies. NMII crosslinks and slides actin filaments past each other, contracting them into actomyosin filament bundles (Fig. 1). Through interaction with adhesion complexes, these actomyosin filament bundles generate forces capable of deforming the cell membrane and associated substrate, and that have underlying functions in processes as diverse as cell migration and neuronal synaptogenesis (reviewed in Vicente-Manzanares et al., 2009a,b). These contractile forces not only shape the cell, they can also be converted into biochemical signals by altering the conformation, association and organization of actin-associated protein complexes, leading to downstream signaling changes such as altered gene expression (reviewed in Humphrey et al., 2014). This process of converting mechanical stimuli into biochemical signals is known as mechanotransduction.

Given the crucial functions that it serves in a diverse range of tissues, it is not surprising that NMII also contributes to diverse disease pathologies, including neuronal disorders, cancer and cardiovascular diseases (reviewed in Ma and Adelstein, 2014). It is also an attractive therapeutic target. For example, vasodilators that target myosin to regulate blood pressure are now being used to treat conditions that result from altered NMII activity. One such vasodilator, fasudil, which is used to increase blood flow following stroke, has also been successfully used to improve learning and working memory in Alzheimer's rodent models (Huentelman et al., 2009; Song et al., 2013), as well as neuron survival and motor function in amyotrophic lateral sclerosis (ALS) and Parkinson's disease rodent models (Tönges et al., 2014; Zhao et al., 2015). Additionally, inhibitors of NMII support the efficient production of pluripotent stem cells and the directed differentiation of specific cell types *in vitro* (see Box 1 for a glossary of terms) (Chen et al., 2010, 2014; Kim et al., 2015; Walker et al., 2010).

**Box 1. Glossary**

**Adhesion-dependent cell migration:** during this process, cells adhere to the extracellular matrix (ECM) through integrin-mediated focal adhesions. This attachment transmits forces to the cell interior, where they are balanced by NMII-mediated tension and result in signaling changes.

**Adhesion-independent cell migration:** cells show weak or no interaction with the ECM; however, cortical actomyosin contractility propels cells through ECM fibers, resulting in a fast migratory process.

**Amoeboid-like cell migration:** a fast migration process that relies on actin cytoskeleton and cell contractility; results in the formation of membrane blebs that allow cells to squeeze through confined spaces. Depending on extracellular cues, cells can switch between amoeboid and mesenchymal cell migration.

**Chromosomal passenger complex (CPC):** the CPC consists of aurora B kinase, survivin, borealin and inner centromere protein (INCENP), and regulates mitotic events, including microtubule–kinetochore attachment and cytokinesis.

**Collective cell migration:** two or more cells move as a group owing to the presence of cell–cell junctions, and the migratory output depends on coordinated cytoskeleton dynamics and cell signaling among all cells in the group.

**Copy number variants (CNVs):** these are large deletions or duplications within the genome, of around ≥30 kb in size.

**Dendritic spines:** post-synaptic protrusions that synapse with pre-synaptic axon terminals. They contain a post-synaptic density (PSD) that clusters neurotransmitter receptors and signaling scaffolds adjacent to the pre-synaptic terminal. In response to repeated excitatory stimulation, the size of the spine head and PSD increases, resulting in synaptic strengthening.

**Growth cones:** dynamic actin-enriched structures at the tips of neurites, axons or dendrites that drive their motility toward a desired target. Chemoattractants and repellents steer growth cones.

**Mesenchymal-like cell migration:** a multi-step process that involves the remodeling of the ECM, the formation of cell protrusions and of adhesions to the substrate, the contractility of the cell body, and the detachment of adhesions at the cell's rear. Depending on extracellular cues, cells can switch between mesenchymal and amoeboid cell migration.

**microRNA (miRNA):** a type of small non-coding RNA that regulates gene expression by silencing complementary RNA targets. Typically, miRNAs consist of ∼22 nucleotides.

**Myosin regulatory light chain (MLC/RLC):** the regulatory light chain for NMII, which is encoded by the *MYL9* gene. The phosphorylation of MLC on Ser19 and/or Thr18 increases myosin ATPase activity, resulting in actin bundling and contraction.

**Pluripotent stem cell:** a cell with the potential to self-renew and to differentiate into any cell lineage of the three germinal layers: ectoderm, endoderm and mesoderm.

**Pre-synaptic terminals:** the axonal compartment in contact with a post-synaptic spine. Pre-synaptic terminals contain synaptic vesicles, which release neurotransmitters into the synaptic cleft in response to action potentials.

**Protrusion:** the broad membrane projection that cells extend during migration. They are characterized by nascent adhesions and fast actin polymerization, which pushes the membrane forward. NMII activity within protrusions results in actin retrograde flow and also leads to adhesion maturation.

**Single-cell migration:** cells move individually and the migratory output relies mainly on the intrinsic properties of the migrating cell and the composition of the microenvironment, such as the presence of chemokines and ECM composition.

**Synaptic plasticity:** stimuli-induced changes in neuronal spine morphology that underlie learning and memory formation.

**Synaptopathies:** neuronal disorders that exhibit altered post-synaptic spine morphology and/or density, and include both neurodevelopmental disorders, such as autism, and neurodegenerative disorders, such as Alzheimer's disease.

**Transendothelial migration:** the process by which cells pass through the endothelial barrier. It can occur through remodeling of cell–cell adhesions at the border of two endothelial cells (paracellular) or by passage of the extravasating cell through the endothelial cell body (transcellullar).

This Review focuses on how NMII and its regulatory pathways contribute to various disorders, while also exploring potential therapeutic benefits and limitations of NMII inhibitors in disease treatment and stem cell therapies. Given the multiple roles of NMII in diverse tissues, therapeutically targeting NMII presents challenges, although targeting upstream regulatory pathways can increase specificity for particular biological processes. We thus describe some of the known upstream regulatory pathways involved, highlighting how Rho GTPase and Ca^2+^ signaling pathways activate kinases that regulate NMII in normal conditions and disease pathology. In addition, we discuss evidence for an emerging role of microRNAs (miRNAs; see Box 1) in the regulation of NMII activity in disease. Because both differential NMII expression and regulation can impact disease pathology, the following section discusses the structure of NMII, different NMII isoforms, and how phosphorylation mediates its association with and bundling of actin filaments, resulting in morphological and signaling changes.

## NMII: structure, function and regulation

The multimeric, bipolar structure of NMII determines its ability to crosslink and contract actin filaments (Pollard, 1982). There are three NMII isoforms (A, B and C; see Box 2), which consist of different NMII heavy chains and shared essential and regulatory light chains (ELCs and RLCs; see Box 1) (D'Apolito et al., 2002; Golomb et al., 2004; Simons et al., 1991). The heavy chain is comprised of a globular head domain, which binds both actin and adenosine triphosphate (ATP) (Rayment et al., 1993a,b); a neck region, which binds both the ELC and RLC (Winkelmann et al., 1984); and a tail region, which homodimerizes in a helical fashion (Côté et al., 1984) (Fig. 1). The non-helical end of the heavy chain tail exhibits the most sequence divergence between the three isoforms, and directs their differential subcellular distributions (Hodge et al., 1992; Ronen and Ravid, 2009; Sandquist and Means, 2008), although recent evidence demonstrates that heterotypical complexes form between NMII isoforms particularly during initial cell spreading (Beach et al., 2014). In addition to homodimerization, NMII filaments associate with each other in an anti-parallel fashion, allowing them to crosslink and contract actin filaments during ATP binding and hydrolysis (Ricketson et al., 2010; Turbedsky et al., 2005). The phosphorylation of the RLC on Ser19 and/or Thr18 regulates the conformation and activity of NMII, with phosphorylation of both residues resulting in increased ATPase activity and a corresponding increase in actin association and bundling (Umemoto et al., 1989; Vicente-Manzanares and Horwitz, 2010; Vicente-Manzanares et al., 2008; Yuen et al., 2009). The resulting actomyosin filament bundles that are at the rear of the cell drive directional migration, and also the post-synaptic maturation of dendritic spines in neurons (Hodges et al., 2011; Vicente-Manzanares et al., 2008, 2011) (see Box 1). Owing to the fundamental role of RLCs in regulating NMII activity, the upstream signaling pathways that mediate the phosphorylation and dephosphorylation of Ser19/Thr18, and the RLC kinases and phosphatases involved, are well studied and play important roles in NMII-mediated cellular processes and pathologies (reviewed in Somlyo and Somlyo, 2003). In the following section, we discuss signaling pathways that mediate NMII activation, and focus on specific kinases that promote RLC phosphorylation leading to NMII activation.

**Box 2. NMII isoforms**

**Non-muscle myosin IIA (NMIIA):** the myosin isoform consisting of non-muscle myosin heavy chain IIA (NMHCIIA), encoded by the *MYH9* gene, and regulatory and essential light chains that are shared with other NMII isoforms. In humans, mutations in *MYH9* result in *MYH9*-related disorders, including May-Hegglin anomaly, Fechtner syndrome and Sebastian syndrome (Heath et al., 2001). In migrating cells, NMIIA preferentially localizes to protrusions and the central region of the cell, where it regulates adhesion maturation (Kolega, 1998; Ronen and Ravid, 2009; Sandquist and Means, 2008; Vicente-Manzanares et al., 2008).

**Non-muscle myosin IIB (NMIIB):** the myosin isoform consisting of NMHCIIB, encoded by the *MYH10* gene, and regulatory and essential light chains that are shared with other NMII isoforms. In migratory cells, NMIIB preferentially localizes to the rear of the cell (Vicente-Manzanares et al., 2008). It is also the primary NMII isoform found at synapses in neurons (Ryu et al., 2006).

**Non-muscle myosin IIC (NMIIC):** the most recently discovered non-muscle myosin isoform, consisting of NMHIIC, encoded by *MYH14*, and regulatory and essential light chains that are shared with other NMII isoforms (Golomb et al., 2004). In neurons, NMIIC, along with NMIIB, drives neurite outgrowth (Wylie and Chantler, 2008).

### NMII: activity regulation

Serine/threonine kinases regulate NMII and function downstream of small Rho GTPases, such as Rac, RhoA and Cdc42, and Ca^2+^/calmodulin signaling pathways (reviewed in Somlyo and Somlyo, 2003) (Fig. 1). In their GTP-bound state, Rho GTPases promote downstream signaling through kinases, such as p21-associated kinase (PAK), myotonic dystrophy kinase-related Cdc42-binding kinase (MRCK), RhoA-associated kinase (ROCK) and citron kinase (Chew et al., 1998; Zeng et al., 2000; reviewed in Heasman and Ridley, 2008 and Vicente-Manzanares et al., 2009b). Guanine nucleotide exchange factors (GEFs) activate Rho GTPases by loading GTP onto them, whereas GTPase-activating proteins (GAPs) stimulate GTP hydrolysis, to inactivate Rho GTPase signaling (reviewed in Heasman and Ridley, 2008). Although able to activate NMII, Rac and Cdc42 signaling pathways generally promote actin polymerization, whereas RhoA, through its effector ROCK, activates NMII and drives the formation of actomyosin filament bundles, which are commonly referred to as ‘stress fibers’ (Chrzanowska-Wodnicka and Burridge, 1996; Jean et al., 2013; Wilkinson et al., 2005; reviewed in Heasman and Ridley, 2008). As a result of its ability to dually regulate NMII activity through direct RLC phosphorylation and indirectly through inhibition of myosin light chain phosphatase (MLCP) (Amano et al., 1996; Katoh et al., 2001; Kimura et al., 1996), ROCK serves as a master regulator of NMII activity. ROCK inhibitors are amongst the most promising therapeutics for NMII-related disorders (reviewed in Mueller et al., 2005 and Pan et al., 2013). Ca^2+^/calmodulin-activated kinases, such as myosin light chain kinase (MLCK) and zipper-interacting protein kinase (ZIPK) (Goeckeler and Wysolmerski, 1995; Murata-Hori et al., 1999), also promote the phosphorylation of RLC on Ser19 and Thr18 (Fig. 1), and are increasingly promising therapeutic targets, especially for cancer treatment (Fu et al., 2015; Gu et al., 2006). NMII kinases can also cooperatively regulate actomyosin organization; for example, in fibroblasts, MLCK-mediated NMII activation forms cortical actin bundles, whereas activation by ROCK generates stress fibers (Totsukawa et al., 2000). In addition to phosphoregulation of NMII activity, increasing evidence demonstrates that miRNAs regulate expression of NMII and its associated regulators. In each of the following sections, we will examine how specific kinases and miRNAs regulate NMII activity to contribute to disease pathology, beginning with an emerging role for NMII misregulation in complex neuronal disorders.

## The role of NMII in neuronal disorders

The presence of actomyosin-like proteins in the brain was first described in 1968 (Puszkin et al., 1968), followed by biochemical and ultrastructural evidence for the presence of NMII at synapses (Blitz and Fine, 1974; Korobova and Svitkina, 2010; Morales and Fifková, 1989). However, NMII has emerged only recently in the complex pathologies that contribute to a diverse range of neurological disorders (reviewed in Nadif Kasri and Van Aelst, 2008). These include synaptic disorders, or synaptopathies (see Box 1), which manifest with altered post-synaptic spine morphology and/or density (reviewed in Penzes et al., 2011). These disorders include: neurodevelopmental disorders, such as autism; neurodegenerative disorders, such as Alzheimer's disease; neuronal migration disorders, such as lissencephaly (Tsai et al., 2007); and disorders of impaired process extension, such as axon regeneration following central nervous system (CNS) injuries (Hur et al., 2011). In addition to a direct role in neuronal cell biology, NMII also regulates glia cell function (Beadle et al., 2008; Rusielewicz et al., 2014), integrity of the blood–brain barrier (as discussed later in this Review) (Beard et al., 2014; Srivastava et al., 2013) and microglia activation in neuroinflammation (Janßen et al., 2014). Here, we discuss the role of NMII in brain development and function, its contribution to diverse neuronal disorders, and the potential therapeutic applications of pharmacological inhibitors of NMII for nervous system disorders and injury.

### NMII in synapse development and function

**Fig. 2.**
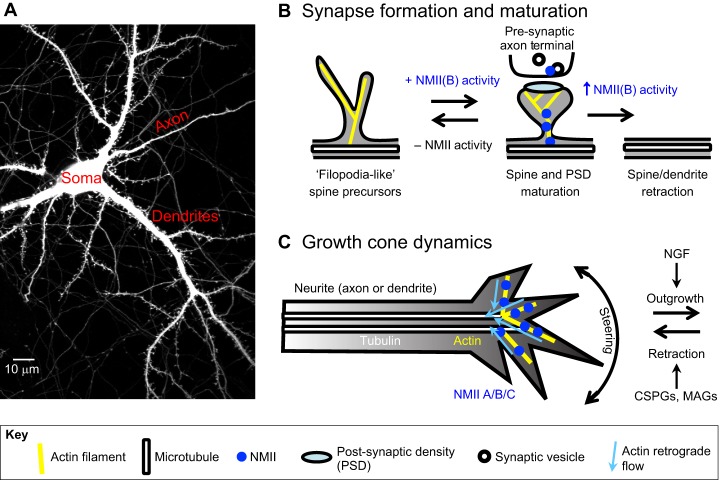
**NMII regulates neuronal plasticity.** (A) Confocal image of a GFP-expressing primary rat hippocampal neuron, highlighting the cell body, or soma, and processes, including post-synaptic dendrites, which form spines, and pre-synaptic axons, which form axon terminals containing synaptic vesicles. (B) NMII drives dynamic changes in neuronal morphology, including changes in dendritic spine formation and maturation, driven primarily by the isoform NMIIB. At the post-synaptic spine, NMII drives changes in actin organization that regulate spine and post-synaptic density (PSD) morphology and size, whereas, on the pre-synaptic side, NMII participates in synaptic vesicle recycling. The absence or inhibition of NMIIB activity results in dynamic ‘filopodia-like’ spine precursors and prevents spine maturation. In contrast, NMIIB activity drives spine and PSD maturation, although further NMIIB activity might result in spine and even dendrite retraction. (C) At the growth cone, all three NMII isoforms are present, and regulate substrate attachment and actin retrograde flow underlying neurite outgrowth. NGF, nerve growth factor; CSPGs, chondroitin sulfate proteoglycans; MAGs, myelin-associated glycoproteins.

In neurons, NMII localizes to dynamic actin-enriched structures, including growth cones (see Box 1) and synapses (Fig. 2). Although neurons express all three NMII isoforms (A, B and C; see Box 2), they are particularly enriched in the NMIIB isoform (Rochlin et al., 1995). Whereas NMIIA, B and C localize to growth cones, where they regulate process extension (Rochlin et al., 1995; Wylie and Chantler, 2008; Wylie et al., 1998), NMIIB is the predominant isoform present at synapses (Ryu et al., 2006). NMIIB localizes both to pre-synaptic terminals (see Box 1), where it mediates synaptic vesicle recycling (Chandrasekar et al., 2013), and post-synaptic dendritic spines (see Box 1), where it regulates the maturation of spines and the clustering of glutamate receptors in the post-synaptic density (PSD) in response to activation of the N-methyl-D-aspartate (NMDA) receptor (Hodges et al., 2011; Rex et al., 2010; Ryu et al., 2006). Thus, NMII serves as an important regulator of stimuli-induced changes in spine morphology that underlie learning and memory formation (reviewed in Hotulainen and Hoogenraad, 2010).

During embryonic development, dendritic spines emerge as dynamic filopodia-like spine precursors (Fig. 2) (Dailey and Smith, 1996; Portera-Cailliau et al., 2003; Ziv and Smith, 1996; reviewed in Yuste and Bonhoeffer, 2004). These spine precursors stabilize upon contact with pre-synaptic terminals (Ziv and Smith, 1996). This results in the formation of thin spines that have a distinct spine head and clustering of glutamate receptors into a signaling scaffold, known as the PSD, at the tip of the spine adjacent to the pre-synaptic terminal (Marrs et al., 2001). In response to further excitatory stimulation, spines mature into a mushroom shape and have a larger PSD, further strengthening the synaptic connection (Fortin et al., 2010; reviewed in Lynch et al., 2007). In the absence of NMII, spines persist as filopodia-like spine precursors with small PSDs and have an impaired ability to respond to excitatory stimulation (Hodges et al., 2011; Rex et al., 2010; Ryu et al., 2006). However, in response to excitatory stimulation, NMII is activated by RLC Ser19, Thr18 di-phosphorylation, resulting in a mature, mushroom-shaped spine and increased PSD size (Hodges et al., 2011).

Serine/threonine kinases regulate myosin activation to drive these changes in spine morphology (Fig. 2). In particular, kinases associated with Rho GTPase signaling pathways are important regulators of synaptic development. For example, RhoA and its effector kinase ROCK promote synaptic strengthening in response to excitatory stimulation by RLC di-phosphorylation, leading to spine maturation (Hodges et al., 2011; Newell-Litwa et al., 2015; Rex et al., 2009). Likewise, the Rho GTPase Rac regulates the formation of dendritic spines and synapses through its effector kinase, PAK, and subsequent RLC phosphorylation (Zhang et al., 2005). Finally, there is some evidence that Ca^2+^/calmodulin-activated MLCK regulates both pre-synaptic vesicle trafficking (Polo-Parada et al., 2005; Tokuoka and Goda, 2006; Yue and Xu, 2014), as well as post-synaptic NMDA receptor currents (Lei et al., 2001). Thus, myosin kinases are vitally important regulators of synaptic strengthening through changes in synapse morphology and molecular composition, which underlie excitatory neurotransmission. In the following section, we will examine how altered Rho GTPase signaling and kinase regulation of NMII contributes to both neuronal and glial disorders, and how therapeutically targeting these pathways alleviates disease symptoms in preclinical models.

### NMII in synaptic disorders

Consistent with the role of NMII and its associated regulatory pathways in synaptic formation and maturation, these pathways have recently emerged as major targets of multiple synaptopathies, including genetically complex neurodevelopmental disorders (Pinto et al., 2010; Zhao et al., 2014). In particular, individuals with non-syndromic mental retardation (MR) exhibit mutations in proteins associated with Rho GTPase signaling pathways that regulate actomyosin activity, including the RhoA GAP, oligophrenin, the Rac GEF, PAK-interacting exchange factor Pix, and the myosin kinase, PAK (reviewed in Ramakers, 2002). Individuals with non-syndromic MR have impaired cognitive ability, with intelligence quotients below 70 (a standard diagnostic marker for intellectual disability) (reviewed in Ramakers, 2002). The majority of non-syndromic MR cases do not exhibit gross anatomical abnormalities, but manifest altered brain ultrastructure, including immature dendritic spines and decreased spine density in adolescence, although fragile X MR results in a persistent increase of immature dendritic spines reminiscent of early development (Irwin et al., 2001; reviewed in Fiala et al., 2002 and Ramakers, 2002). Similarly, schizophrenia is associated with decreased spine density and maturation (Penzes et al., 2011), whereas autistic individuals frequently exhibit increased spine density (Hutsler and Zhang, 2010; reviewed in Penzes et al., 2011). A recent study of *de novo* mutations found in multiple neurodevelopmental disorders revealed that *MYH9* (Box 2) is one of only three affected genes shared by autism, schizophrenia and intellectual disability, and *de novo* mutations for *MYH10* (Box 2) are reported for both schizophrenia and autism (Li et al., 2015b). Additionally, pathways that regulate NMII function, especially Rho GTPase signaling pathways, are disproportionately targeted by autism and schizophrenia copy number variants (CNVs; see Box 1) (Pinto et al., 2010; Zhao et al., 2014). Whether these *de novo* mutations and CNVs in NMII and NMII regulatory pathways contribute to disease progression still needs to be established. However, recent evidence demonstrates that altered NMII regulation contributes to disease pathology in Timothy syndrome, which lies on the autism spectrum, through RhoA-mediated NMII activation leading to dendrite retraction (Krey et al., 2013).

How might these copy number variations that are found in NMII signaling pathways contribute to synaptic abnormalities in neurodevelopmental disorders? Although the mechanism is unknown, altered expression of NMII regulatory proteins could lead to abnormal NMII activation, particularly at critical periods of brain development that involve synapse formation and synaptic pruning. For example, increased NMII activation might underlie the reduced synaptic density that features in schizophrenia by preventing the formation of spine precursors that occurs in the absence of NMII activity (Hodges et al., 2011; Ryu et al., 2006) or by promoting spine retraction. Consistent with this hypothesis, increased levels of phosphorylated RLCs have been observed in the anterior cingulate cortex of brains from schizophrenics (Rubio et al., 2012).

Although NMII inactivation promotes the formation of spine precursors (Hodges et al., 2011; Ryu et al., 2006), subsequent NMII activation is necessary for spine maturation and stabilization (Hodges et al., 2011; Zhang et al., 2005). Thus, decreased NMII activity could account for the decreased spine density observed in individuals with non-syndromic MR. Consistent with this hypothesis, inactivation of the non-syndromic MR-related NMII kinase PAK in rat hippocampal neurons resulted in decreased spine density, but this phenotype was rescued by myosin activation through the co-expression of a phosphomimetic RLC (Zhang et al., 2005). Alternately, decreased spine density could arise from increased NMII contractility during spine formation or through elevated contractility leading to spine retraction and synapse pruning. In support of this hypothesis, the knockdown of the non-syndromic MR-related protein oligophrenin in rat hippocampal slices resulted in decreased spine density and/or length, which could be rescued by inhibition of myosin activity (Govek et al., 2004; Nadif Kasri et al., 2009). Thus, insights from known non-syndromic MR disease targets indicate that the mechanism that underlies similar synaptic deficits will likely depend on the affected molecule(s) and when/where they are active during brain development. Further studies are needed to elucidate how actomyosin pathways are regulated both temporally and spatially to determine spine morphology and density at distinct stages of brain development. Furthermore, in genetically complex disorders, such as autism and schizophrenia, studies are needed to elucidate the contribution of specific CNV-associated genes, which is now possible through the use of gene editing technologies in model organisms (Swiech et al., 2014).

In addition to genetic alterations in the components of NMII-related signaling pathways, miRNAs have recently emerged as regulators of actomyosin signaling pathways that affect brain development and disease. For example, miR-137, which suppresses PAK signaling (Liu et al., 2011), associates with schizophrenia (Ripke et al., 2011). In autism, differentially expressed miRNAs disproportionately target actomyosin regulatory pathways (Mundalil Vasu et al., 2014). In Down syndrome, miR-155 is upregulated, leading to synaptic dysfunction through downregulation of sorting nexin 27 (SNX27) (Lu et al., 2013; Wang et al., 2013a), although miR-155 can also suppress NMII activation (Weber et al., 2014), highlighting that miRNAs often regulate multiple targets that could function in disease pathology. Thus, although miRNAs could serve as clinical therapeutic targets for multiple neurological disorders, further investigation into their specificity for actomyosin pathways contributing to disease pathology is necessary.

### NMII regulators as therapeutic targets

NMII kinases are emerging as attractive therapeutic targets for the treatment of diverse synaptopathies (Table 1). For example, PAK inhibitors successfully reverse abnormal spine morphology in animal models of both fragile X MR (Dolan et al., 2013) and schizophrenia (Hayashi-Takagi et al., 2014). Likewise, ROCK inhibitors, which promote neurite outgrowth to restore neuronal connectivity, could be especially useful for the treatment of neurodegenerative disorders, such as Parkinson's disease and Alzheimer's disease, for which they are currently undergoing preclinical trials in animal models (Couch et al., 2010; Huentelman et al., 2009; Tatenhorst et al., 2014; Tönges et al., 2012; Zhao et al., 2015). In addition to neurodegenerative disorders, ROCK inhibitors are being explored as a mechanism to treat altered synaptic connections and behavior that result from drug addiction (DePoy et al., 2013; Roland et al., 2014). Thus, myosin regulatory kinases represent attractive therapeutic targets for diverse synaptic disorders. However, off-target effects of kinase inhibitors often prevent systemic application, thus necessitating mechanisms for localized delivery. For example, in addition to their effects on spine morphology, ROCK inhibitors affect blood pressure and permeability of the blood–brain barrier (Huang et al., 2011). Moreover, the NMII inhibitor blebbistatin (Fig. 1) inhibits both muscle and non-muscle myosin II, and thus affects both skeletal and cardiac muscle contractions (Dou et al., 2007; Stewart et al., 2009). These examples highlight the need for more specific inhibitors and identification of alternate targets for perturbing NMII activity specifically within the desired tissue.

**Table 1.**
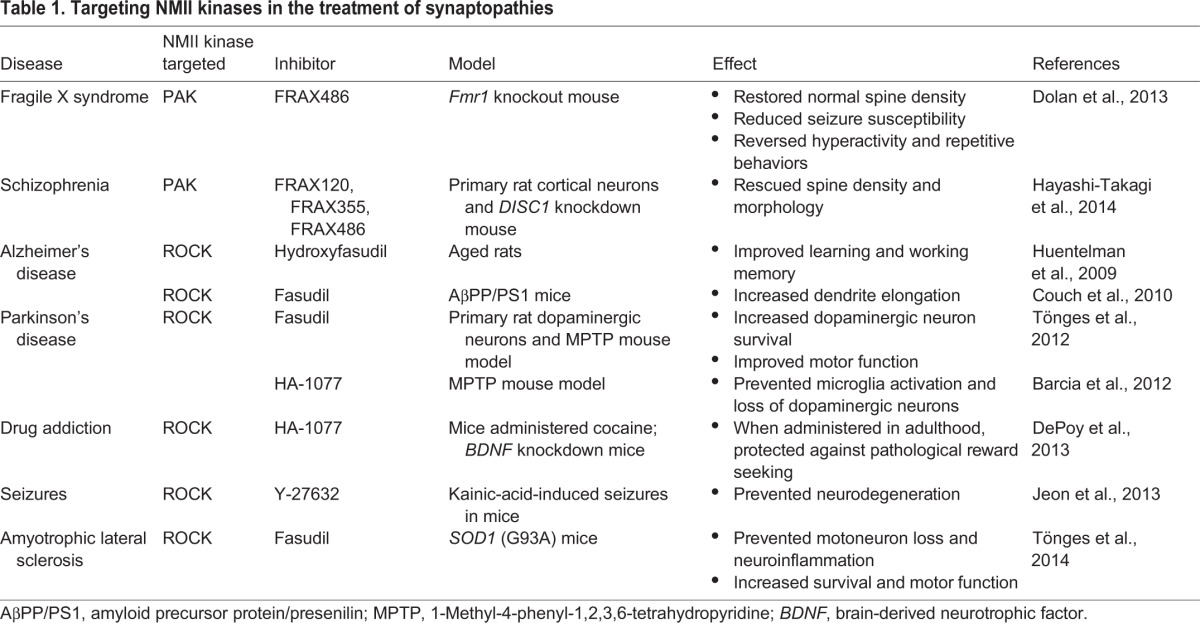
**Targeting NMII kinases in the treatment of synaptopathies**

### NMII in neuronal regeneration following CNS injury

Unlike synapses, where NMIIB is the predominant isoform, growth cones express all three NMII isoforms (Rochlin et al., 1995; Wylie and Chantler, 2008). In a neuroblastoma Neuro2A model of neurite extension, NMIIB and NMIIC promote neurite outgrowth, whereas NMIIA and NMIIC promote adhesion to the fibronectin substrate (Wylie and Chantler, 2001, 2008; Wylie et al., 1998) (Fig. 2). Whereas *NMIIA* expression remains constant before and during neurite growth, *NMIIB* mRNA levels increase during neurite outgrowth (Wylie et al., 1998). During development, NMII-mediated actin remodeling drives axonal extension and retraction in response to attractants, such as nerve growth factor (NGF), or to repellents, such as netrin-1 and semaphorin-3A, to refine the innervation of the correct brain targets (Brown and Bridgman, 2009; Brown et al., 2009; Loudon et al., 2006; Murray et al., 2010; Myers et al., 2006).

Following CNS injury, some of the repellents that refine neural circuitry during development, such as myelin-associated glycoproteins (MAGs) and chondroitin sulfate proteoglycans (CSPGs), inhibit axon regeneration and re-innervation within the glia scar (Fig. 2) (reviewed in Busch and Silver, 2007). The removal of these inhibitory factors has met with limited therapeutic success, suggesting that adult axons must overcome intrinsic factors that prevent their growth into an injury site (reviewed in Wootla et al., 2012). RhoA/ROCK-mediated NMII activation functions downstream of inhibitory CSPGs, and the ROCK inhibitor Y-27632 partially rescued axon regrowth in an *in vivo* rat spinal cord injury model and *in vitro* in chick dorsal root and retinal ganglion cells as well as rat cerebellar granule neurons (Borisoff et al., 2003; Kubo et al., 2008; Monnier et al., 2003). However, blebbistatin restored, and even surpassed, control axon growth on inhibitory substrates *in vitro* (Hur et al., 2011; Kilinc et al., 2014; Yu et al., 2012). These pharmacological differences could be due in part to the observation that the ROCK inhibitor Y-27632 leads to increased CSPGs within the glia scar in a rat spinal cord injury model (Chan et al., 2007). Thus, NMII is emerging as an attractive therapeutic target for axon regeneration in debilitating spinal cord injuries and CNS lesions.

### NMII in glia cell biology

Besides neurons, NMII is also involved in the biology of glia cells. Three glia cell types reside in the CNS: astrocytes, oligodendrocytes and microglia. Astrocytes are the predominant glial cell type in the brain, where they serve multiple functions, including the regulation of blood flow and glutamate uptake at synapses (reviewed in Rossi, 2015). A branched, stellate morphology allows astrocytes to interact with these multiple targets, including the endothelium of the blood–brain barrier and synapses between neurons (reviewed in Rossi, 2015). The inactivation of ROCK-mediated NMII activation is required for this branched morphology (Murk et al., 2013; Ramakers and Moolenaar, 1998), and is also responsible for the reactive astroglial phenotype observed in response to interleukin (IL)-1β, the cytokine that mediates astrocyte scar formation following CNS injury (John, 2004). However, because the directed migration of astrocytes toward the site of injury requires NMII (Peng et al., 2013), ROCK inhibition could be used to prevent glia scar formation (Lau et al., 2012). Thus, in addition to promoting axon regrowth into the injured site, NMII inhibition can also help prevent glial scar formation.

Astrocytes are also responsible for clearing glutamate from the synaptic cleft, a process mediated by the excitatory amino acid transporters (EAATs) (reviewed in Jensen et al., 2015). ROCK-mediated myosin contractility regulates glutamate uptake by determining the astrocyte surface expression of EEAT1/2 (Lau et al., 2011). Several neuronal disorders present with increased glutamate, resulting in cytotoxicity and/or abnormal neurotransmission (Nakagawa and Kaneko, 2013). For example, increased glutamate lowers the seizure threshold in epilepsy, although ROCK inhibition protects neurons from the resulting cytotoxicity following a seizure (Jeon et al., 2013). Thus, the myosin kinase ROCK is a promising therapeutic target for disorders associated with glutamate misregulation.

NMII also regulates myelination, which enables the rapid conductance of action potentials. In the CNS, oligodendrocytes branch to ensheath multiple axonal targets. During the process of oligodendrocyte differentiation and branching in rodent *in vitro* models, NMII is downregulated, with NMII activity preventing oligodendrocyte differentiation and NMII inhibition enhancing oligodendrocyte maturation and myelination (Wang et al., 2008, 2012a). NMII inhibition promotes re-myelination following brain lesion in mice (Rusielewicz et al., 2014). In contrast to oligodendrocytes, Schwann cells in the peripheral nervous system require NMII to elongate on and ensheath axons (Wang et al., 2008), with robust ROCK-driven myosin phosphorylation characterizing the onset of myelination (Melendez-Vasquez et al., 2004). Thus, pharmacological modulators of NMII activity could potentially be used to promote myelination in demyelinating disorders, such as multiple sclerosis.

Finally, NMII contributes to microglia function and to neuroinflammation resulting from microglia activation and release of inflammatory cytokines (reviewed in Schwartz et al., 2013). In a mouse model of Parkinson's disease, ROCK inhibition prevents microglia activation and the phagocytosis of degenerating dopaminergic neurons (Barcia et al., 2012). Similarly, ALS results in increased pro-inflammatory cytokine production, which is attenuated by ROCK inhibition, leading to increased mouse motoneuron survival *in vivo* (Ding et al., 2010; Parisi et al., 2013; Tönges et al., 2014). As in synaptopathies, NMII-regulatory miRNAs might contribute to the pathology of neuroinflammatory disorders. In line with this, miRNAs associated with myosin regulation are upregulated in ALS (Parisi et al., 2013). In multiple sclerosis, miR-155, which is known to regulate NMII activity (Weber et al., 2014), promotes inflammation (Moore et al., 2013). Thus, a detailed survey of myosin regulatory miRNAs in neuronal and glia function, particularly at discrete stages of development, would be greatly informative. In the following section we will address how NMII regulation, which shapes neuronal and glial functions, similarly drives morphological and signaling changes associated with cancer cell division and migration.

## NMII in cancer

Several cancers exhibit differential expression and/or activation of NMII isoforms and their associated regulators (Table 2), leading to changes in cell division and migration that underlie tumorigenesis and invasion. Both oncogenes and miRNAs regulate this differential NMII expression, although external factors within the tumor microenvironment, such as the extracellular matrix (ECM) and cytokines, also profoundly influence NMII activity. Understanding how intrinsic genetic factors and external factors within the tumor environment combine to regulate NMII expression/activity could allow for therapeutic intervention at distinct stages of cancer progression.

**Table 2.**
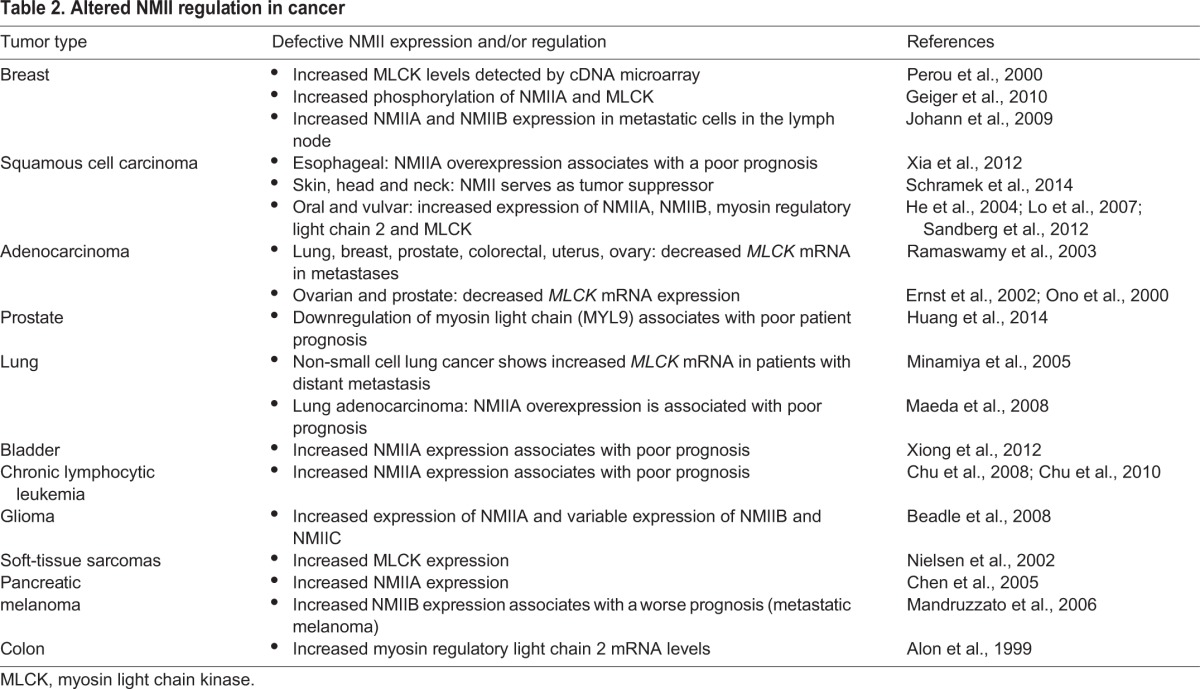
**Altered NMII regulation in cancer**

### NMII and tumorigenesis

During tumorigenesis, mutations in oncogenes and/or in tumor suppressor genes result in uncontrolled cell division, decreased apoptosis, and changes in cell differentiation and motility. Several oncogenes activate NMII to promote these tumorigenic properties. For example, the oncogenes *Ras* and its downstream target *BRAF*, a serine-threonine kinase, which regulate cell survival and proliferation through a mitogen-activated protein kinase (MAPK) signaling cascade, also increase NMII activation, leading to tumor invasion *in vitro* and in a mouse melanoma model *in vivo* (Arozarena et al., 2011; Chen et al., 2003; Helfman and Pawlak, 2005; Zhong et al., 1997). Conversely, some tumor suppressors downregulate NMII-mediated contractility *in vitro*. The tumor suppressor p53 decreases RhoA activation and also alters NMIIB expression, both resulting in impaired tumor invasion (Xia and Land, 2007; Yam et al., 2001). Interestingly, Schramek et al. (2014) demonstrated that NMIIA acts as a tumor suppressor, with NMIIA downregulation resulting in impaired activation of p53 in keratinocytes both *in vitro* and *in vivo*. Additionally, non-coding miRNAs might directly and/or indirectly target NMII and its regulators, resulting in changes to tumor cell migration and proliferation (Table 3). Taken together, these findings suggest that diverse carcinogenic mutations act on the expression and activity of NMII at the onset of tumorigenesis and later on during invasion, implicating NMII regulation in the pathogenesis of multiple, different tumor types. Below, we will specifically examine how this altered NMII activity contributes to cancer progression through the regulation of cell division and cancer cell migration/metastasis.

**Table 3.**
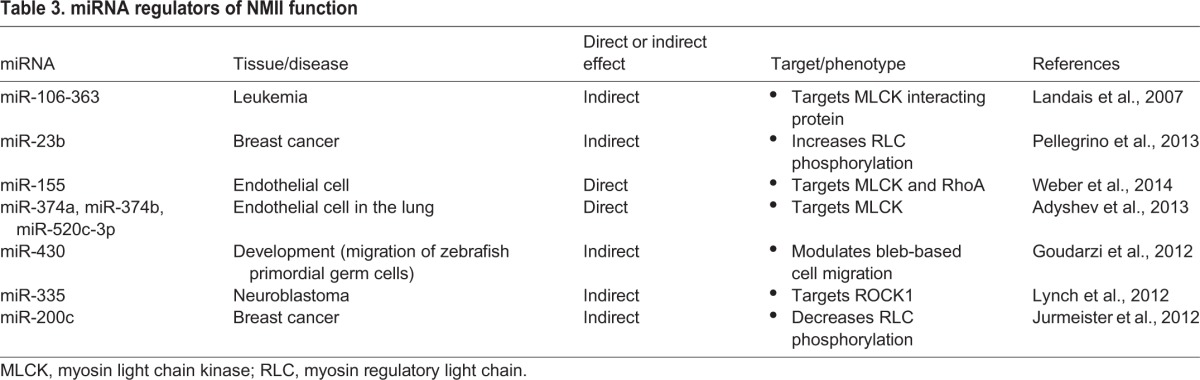
**miRNA regulators of NMII function**

### NMII in cell division: propagating genetic abnormalities

**Fig. 3.**
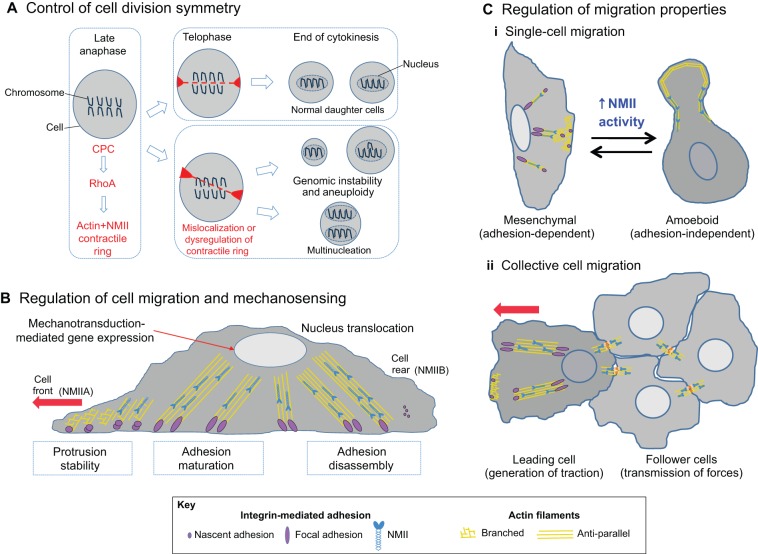
**NMII drives cancer cell progression.** (A) NMII in cell division. At the end of anaphase during mitosis, the chromosomal passenger complex (CPC) induces RhoA-mediated formation of an actin-NMII contractile ring (red), resulting in cytokinesis and division into two daughter cells (upper box; telophase). However, mislocalization of the contractile ring (lower box) can result in genetic abnormalities that are linked to cancer, such as aneuploidy or multinucleation. (B) NMII in cell migration and metastasis. During adhesion-dependent cell migration (see Box 1), cells polymerize actin at the cell front (unaligned yellow lines), while integrin-based adhesions (purple circles) mediate attachment to the extracellular matrix. NMIIA (blue) generates forces that alter actin organization at the cell front and initiate adhesion maturation (purple ellipses indicate adhesion elongation). NMIIB (blue) is involved in the formation of stress fibers (aligned yellow lines), nucleus translocation and in the detachment of adhesions at the cell rear. These mechanical forces induced by NMII can also influence other biochemical pathways (mechanotransduction) that are able to modify cell behavior. (C) NMII participates in various modes of cell migration. (i) Invasion of a single tumor cell is either mesenchymal (adhesion-dependent) or amoeboid (adhesion-independent). For mesenchymal-like, single-cell migration, myosin regulates the migration process as described in B; for amoeboid-like single-cell migration, increased NMII-mediated tension affects the cortical actin network (Ruprecht et al., 2015), allowing for adhesion-independent migration through porous matrices. (ii) During collective cell migration, the leading cell generates NMII-mediated traction forces that are propagated to the follower cells through cell–cell adhesions (see Fig. 4 for details).

During cytokinesis, actomyosin filaments organize into a contractile ring that separates dividing daughter cells (reviewed in Barr and Gruneberg, 2007). The chromosomal passenger complex (CPC; see Box 1) positions RhoA activity within the cytokinetic ring, resulting in localized NMII forces that drive cell division (DeBiasio et al., 1996; Ou et al., 2010; Yang et al., 2012). Even after failed cytokinesis, NMII-mediated traction forces resolve binucleate cells into euploid progeny in interphase (Choudhary et al., 2013). However, in cancer cells, the altered localization and regulation of NMII activity can induce genetic abnormalities, such as aneuploidy (Fig. 3A). For example, in breast cancer cell lines, the downregulation of the tumor suppressor gene *BRCA2 in vitro* results in NMII mislocalization during mitosis, leading to chromosome instability and aneuploidy (Daniels et al., 2004; Takaoka et al., 2014). The NMIIC isoform is associated with delayed cytokinesis in lung tumor cells (Jana et al., 2006), whereas several tumor cell lines exhibit decreased RLC phosphorylation and increased multinucleation, likely resulting from failed cytokinesis (Wu et al., 2010). Thus, the correct regulation of NMII activity and its positioning within the cytokinetic ring preserves genome integrity. How genetic abnormalities arising from dysregulated NMII contribute to disease pathogenesis requires further investigation. However, NMII-mediated cell division can also coordinate tumor cell invasion by promoting cell detachment and facilitating the rupture of the epithelial basement membrane (Vasiliev et al., 2004), thus serving as a master regulator of cancer progression.

### NMII in tumor invasion and metastasis

In addition to cell cycle regulation, NMII drives several key steps that are necessary for tumor invasion and metastasis (Fig. 3B,C), making it an attractive chemotherapeutic target. Cancer cells exhibit diverse migratory behaviors, depending on tumor type and localization (reviewed in Friedl and Alexander, 2011). These include single versus collective cell migration (Fig. 3C), and adhesion-dependent versus -independent migration (see Box 1; also reviewed in Friedl et al., 2012). In all cases, NMII is emerging as an important regulator of tumor metastasis. During adhesion-dependent, mesenchymal-like single-cell migration (see Box 1), the localized activity of distinct NMII isoforms determines the speed and persistence of migration *in vitro*. For example, NMIIA localizes to protrusions (see Box 1) at the front of the cell, where it regulates adhesion maturation, whereas NMIIB forms the contractile rear and drives adhesion maturation, nucleus dislocation and detachment from the substrate, thus propelling the cell in a forward direction (Gomes et al., 2005; Vicente-Manzanares et al., 2007). In adhesion-independent, amoeboid-like single-cell migration (see Box 1), NMII-mediated contractility allows normal cells (such as fibroblasts and zebrafish progenitor cells) or tumor cells (human melanoma and adenocarcinoma) to invade porous matrices *in vitro* without the need for ECM remodeling by generating forces that influence actin cortical flow to propel cells forward (Petrie et al., 2012; Ruprecht et al., 2015; Sahai and Marshall, 2003). For example, gliomas use NMII-dependent contractility to squeeze through submicrometer pores within the brain*,* and inhibition of NMII activity by blebbistatin prevents glioma invasion *in situ* and *in vitro* in response to diverse pro-migratory signals (Beadle et al., 2008; Salhia et al., 2008). Experiments *in vitro* and *in vivo* demonstrate that, during collective tumor cell migration (see Fig. 3C), NMII promotes traction forces at the leading edge of cells, which drags the follower cells and generates a supra-cellular mechanical organization that contributes to the migration process (Cai et al., 2014; Gaggioli et al., 2007; Ng et al., 2012; Reffay et al., 2014). NMII-mediated contractility also influences the transition between collective and single-cell migration that is observed during epithelial-to-mesenchymal transition underlying tumor invasion, by exerting forces that accelerate the turnover of cell–cell junctions (Peglion et al., 2014). Because NMII plays a central role in diverse migratory behaviors, the pharmacological manipulation of NMII might be considered as a complementary therapeutic tool for improving the success of cancer therapies, especially in the treatment of highly metastatic tumors.

NMII activity also facilitates migration by enabling tumor cells to respond to both physical and biochemical cues within the microenvironment (Friedland et al., 2009; Meshel et al., 2005; Zhong et al., 1998). The tumor microenvironment is a complex structure composed of ECM components, such as collagen, fibronectin and laminin, as well as multiple, abnormally expressed growth factors and chemokines that combine to regulate myosin activity (Asokan et al., 2014; Dulyaninova et al., 2007; Harrison et al., 2013; Kharait et al., 2006; Klemke et al., 1997; Liu et al., 2014; Nakashima et al., 2011; Shinto et al., 2010; Straussman et al., 2001; Zhou et al., 2008). The physical stiffness of the tumor microenvironment positively correlates with NMII-mediated contractility, and increased contractility facilitates migration on stiffer substrates, as observed in bone metastasis (Liu et al., 2009). Interestingly, cancer-associated fibroblasts exhibit increased NMII activity, resulting in ECM fiber realignment and facilitating tumor invasion of connective tissue (Calvo et al., 2013; Yamaguchi et al., 2014). Furthermore, in mechanotransduction, physical forces generated by NMII alter the adhesion properties and downstream signaling pathways that regulate diverse cellular events, including cell proliferation, apoptosis and gene expression (reviewed in Humphrey et al., 2014). For example, glioma cells exhibit increased proliferation and cell migration *in vitro* on stiffer substrates owing to changes in NMII-related signaling (Ulrich et al., 2009). Thus, NMII not only serves as a mechanical regulator of cell migration, but also as a pivotal regulator of biochemical signaling that results from changes in adhesion composition and that influences several hallmarks of cancer (reviewed in Hanahan and Weinberg, 2011).

In addition to the physical stiffness of the tumor microenvironment, soluble growth factors and cytokines can also influence NMII activity and thus the behavior of cancer cells. In prostate cancer, the activation of protein kinase C delta (PKCδ) by epidermal growth factor (EGF) increases the phosphorylation of NMIIB (Kharait et al., 2006; Straussman et al., 2001). Similarly, signaling by EGF results in the increased phosphorylation of NMIIA in both breast and pancreatic tumor cell lines *in vitro* (Dulyaninova et al., 2007; Nakashima et al., 2011). The association of hepatic growth factor (HGF) and EGF induces MLCK activation in breast cancer cells, resulting in increased proliferation and migration of the tumor cells (Harrison et al., 2013). In gastric cancer, transforming growth factor β (TGFβ) induces RhoA activation and RLC phosphorylation, increasing tumor migration speed and invasiveness (Shinto et al., 2010). In addition, there are reports of cross-talk between myosin regulatory proteins and mitogen-activated pathways (Klemke et al., 1997; Zhou et al., 2008). There is also *in vitro* evidence that interleukins and chemoattractants can modulate myosin activation under physiological conditions (Asokan et al., 2014; Liu et al., 2014; Rah et al., 2007). The contribution of immune cells and inflammation to tumor cell behavior (reviewed in Hanahan and Weinberg, 2011) warrants investigation into how abnormal IL expression affects NMII activity in cancer disease pathology. As discussed below, vascular biology might provide mechanistic insights into how inflammatory cytokines regulate tumor behavior, because pro-inflammatory cytokines can increase NMII activity in endothelial cells, leading to the rupture of adherens junctions (Huppert et al., 2010; Weidert et al., 2014).

Conserved roles for NMII in cancer progression of diverse tumor types has led to the development of novel chemicals that regulate NMII activity, many of which show promising results *in vitro* (Table 4). Here, we have addressed how NMII promotes cancer by generating genetic abnormalities and driving cell migration, although NMII activity might also contribute to other stages of cancer, such as blood vessel recruitment (discussed below). These multiple roles in cancer progression make NMII an attractive chemotherapeutic target.

**Table 4.**
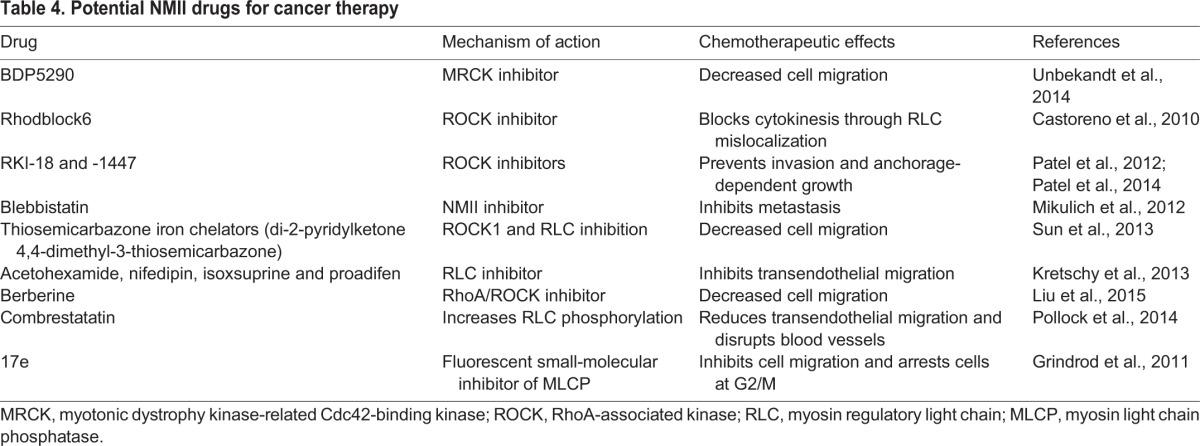
**Potential NMII drugs for cancer therapy**

## Myosin and vascular diseases

Myosin-related contractility plays an important role in the maintenance of blood vessel integrity. The vascular system consists of arteries, veins and capillaries. While capillaries are composed of an endothelial cell monolayer, which expresses NMII, arteries and veins consist of three cell layers: the intima layer, compromised of NMII-expressing endothelial cells; the muscular layer, containing both NMII and smooth muscle myosin; and the adventitial layer, which consists of connective tissue that contains fibroblasts expressing NMII. Endothelial cells throughout the vascular system are exposed to shear force fluctuations from blood flow. In response to these forces, NMII mediates changes in actomyosin organization and signaling that regulate endothelial cell–cell adhesions and the resulting vascular permeability (Conway et al., 2013; Tornavaca et al., 2015). However, both chronic and acute vascular events, such as atherosclerosis and edema, compromise NMII activity in endothelial and smooth muscle cells, leading to drastic changes in blood vessel homeostasis and contributing to the development of vascular pathologies. In this section, we address the main roles of NMII in blood vessel integrity and how NMII expression/function is deregulated during the development of vascular diseases.

### Myosin and vascular permeability

**Fig. 4.**
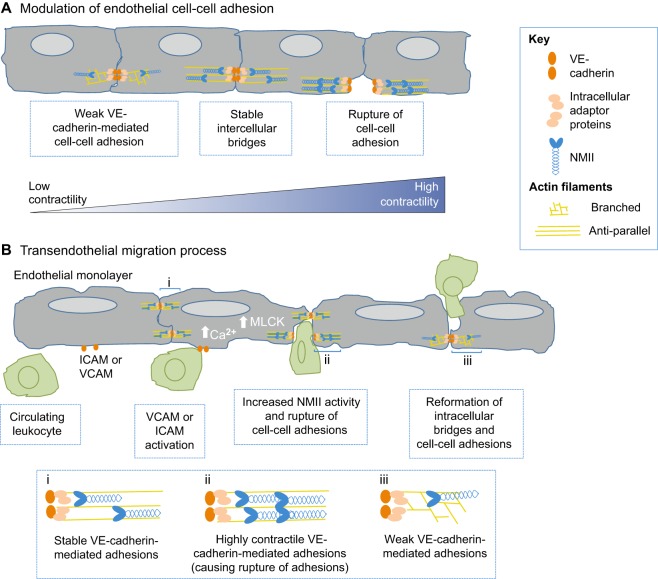
**NMII** **regulates vascular biology and disease.** (A) NMII remodels endothelial adherens junctions. VE-cadherin (orange ovals) mediates intercellular links between neighboring cells. Intracellularly, catenins (beige ovals) link VE-cadherin with the actin cytoskeleton (yellow lines). Myosin (blue) generates forces that stabilize intercellular bridges. However, enhanced NMII-mediated contractility can lead to the rupture of cell-cell adhesions. (B) NMII facilitates transendothelial migration. During rolling, leukocytes (green) interact with ICAM or VCAM receptors at the membrane of endothelial cells, resulting in an influx of Ca^2^^+^. At the adherens junctions at the cell border, increased NMII activity mediated by MLCK results in the rupture of cell–cell adhesions. After leukocytes squeeze and pass by the border of the two endothelial cells (paracellular), there is a neo-formation of VE-cadherin. ICAM, intercellular adhesion molecule; VCAM, vascular cell adhesion molecule; MLCK, myosin light chain kinase.

The permeability of the blood barrier regulates the flow of cells and molecules between intravascular and extravascular compartments (reviewed in Mehta and Malik, 2006). This paracellular permeability relies on proper cell–cell adhesions, which are regulated by NMII (Fig. 4A). In endothelial cells, adherens junctions are mediated by vascular endothelial cadherin (VE-cadherin), a member of the cadherin family of transmembrane receptors, which interacts with the actin cytoskeleton network through adaptor proteins, including β-catenin and p120-catenin (reviewed in Giannotta et al., 2013). NMIIA and NMIIB isoforms participate in the formation of actin bundles at the interdigitating filopodia-like structures that are observed during the formation of both VE-cadherin- and epithelial-cadherin (E-cadherin)-mediated intercellular bridges (Hoelzle and Svitkina, 2012; Smutny et al., 2010; reviewed in Yonemura, 2011). However, whereas NMII normally stabilizes adherens junctions, increased NMII-mediated contractility can disrupt endothelial junctions, as observed during the transendothelial migration of leukocytes (see Box 1; reviewed in Muller, 2011). Inflammatory factors, such as interleukins, nitric oxide (NO) and oxidative stress, increase Ca^2+^ influx and MLCK-mediated endothelial cell contractility, disrupting cell–cell adhesions and increasing the permeability of microvessels (Cromer et al., 2014; Gandhirajan et al., 2013; Liao et al., 2013; Mishra et al., 2015; Rees et al., 2012; Shen et al., 2010). Thus, the tight regulation of NMII activity maintains vascular integrity, whereas its misregulation results in barrier dysfunction in acute vascular pathologies, such as the accumulation of interstitial liquid that occurs in edema, and in chronic diseases, such as inflammation and cancer (discussed below).

NMII activity regulates endothelial cell–cell junctions, facilitating transendothelial leukocyte migration (Fig. 4B). During leukocyte rolling, the activation of vascular cell adhesion molecule 1 (VCAM-1) and intercellular adhesion molecule 1 (ICAM-1) on endothelial cells increases cytoplasmic Ca^2+^ levels, resulting in MLCK-mediated contractility and a consequent rupture of cell–cell adhesions (Haidari et al., 2011; Lorenzon et al., 1998; van Buul et al., 2007, 2010; reviewed in Muller, 2011) (Fig. 4B). Leukocytes also release inflammatory cytokines, such as IL-4, that increase endothelial RLC phosphorylation, disrupting cell junctions and allowing for transendothelial migration (Haidari et al., 2011; Lorenzon et al., 1998; van Buul et al., 2007, 2010; reviewed in Muller, 2011). As previously discussed, NMII activity facilitates leukocyte nuclear compression, allowing cells to traverse through endothelial cell–cell junctions (Jacobelli et al., 2013; Manes and Pober, 2013; Stroka et al., 2013). Interestingly, CD4+ T cells from elderly individuals exhibit decreased NMIIA expression and defective transendothelial migration (Cane et al., 2012). Thus, NMII regulates immune response and inflammation by remodeling endothelial cell–cell adhesions, allowing for leukocyte invasion. Consistent with a role for NMII in inflammation, the NMII inhibitor blebbistatin decreases inflammation in the rat kidney *in vivo* by blocking leukocyte infiltration (Si et al., 2010).

Changes in endothelial permeability and leukocyte migration might also contribute to atherosclerosis, in which chronic inflammation of the endothelial layer results in plaques containing lipids and minerals that obstruct arteries and perturb blood flow, and, when ruptured, can lead to blood vessel occlusion (reviewed in Galkina and Ley, 2009). These plaques are commonly formed in regions of bifurcation of large and medium arteries, which are characterized by disturbances in blood flow (reviewed in Davies et al., 2013). A possible explanation for this coincidence is that localized perturbations in fluid shear stress result in NMII-mediated mechanotransduction signals that modulate the behavior of, and patterns of gene expression in, endothelial cells (reviewed in Hahn and Schwartz, 2009). Consistent with this hypothesis, NMIIB and its associated RLCs are increased in atherosclerotic plaques (de la Cuesta et al., 2011; Nikol et al., 1997), and oxidized phospholipids associated with atherosclerosis increase RhoA-driven myosin activity (Essler et al., 1999; Zimman et al., 2010). The pharmacological inhibition of myosin kinases, either MLCK or ROCK, have been found to reduce the development and progression of atherosclerotic plaques in mouse models of atherosclerosis (Wu et al., 2009). Chronic upregulation of myosin activity in atherosclerosis likely increases endothelial permeability as previously discussed, resulting in localized inflammation in the intima vascular layer as observed during the early development of atherosclerosis (Haidari et al., 2011).

These differential effects of NMII activity on endothelial cell–cell junctions are also observed in cancer metastasis, where tumor cells increase endothelial NMII activity, disrupting adhesions and allowing for transcellular migration and tissue invasion (Khuon et al., 2010; Li and Zhu, 2015; Li et al., 2015a). For example, melanoma cells increase endothelial cell contractility *in vitro*, leading to the rupture of cell–cell adhesion by the simultaneous activation of VCAM and IL1β- and IL8-mediated signaling pathways (Weidert et al., 2014). Interestingly, although better known for triggering microtubule destabilization, the anti-angiogenic drug combretastatin (Table 4) also regulates RLC phosphorylation in endothelial and T cells, disrupting T-cell translocation (Kanthou and Tozer, 2002; Nathan et al., 2012; Pollock et al., 2014). Thus, NMII is a promising therapeutic target for conditions of exacerbated transendothelial migration, such as cancer and chronic inflammation, because of its dual roles in endothelial adhesion and migration of the invading cell.

### NMII in angiogenesis and disease therapy

**Fig. 5.**
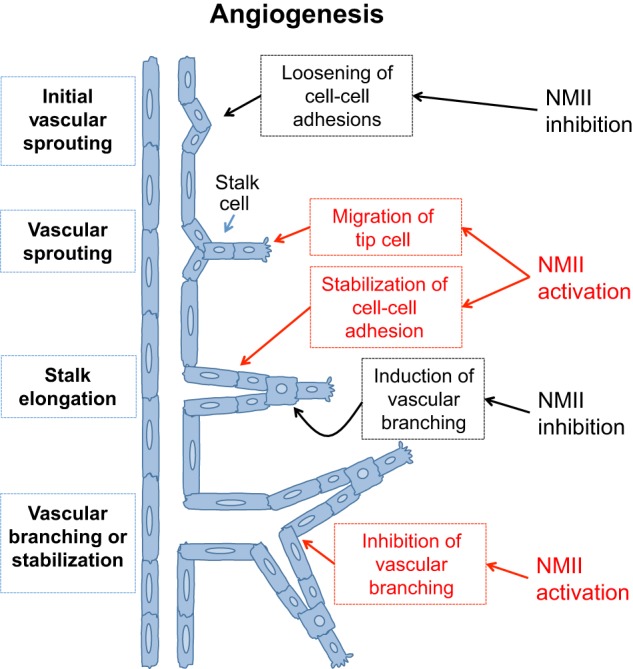
**Differential NMII activity defines distinct stages of angiogenesis.** After the detection of stimuli (hypoxia or growth factors), NMII inhibition results in the loosening of endothelial adherens junctions, favoring the detachment of migrating endothelial cells (tip cell). The tip cell migrates into the connective tissue, in a mechanism that relies on NMII activation (see Fig. 3B). During collective cell migration (stalk elongation), NMII activation stabilizes the junctions of the follower endothelial cells, resulting in stalk elongation. During stalk elongation, the localized activation or inhibition of NMII results in the inhibition or induction of vascular branching, respectively.

During the process of angiogenesis, when new blood vessels form from existing vessels, NMII similarly regulates endothelial cell–cell junctions, while also directing cell–matrix interactions that promote endothelial cell migration. During initial vascular sprouting and vessel branching, the disruption of endothelial cell junctions increases cell interaction with the ECM, facilitating the migration of tip cells (Fig. 5). The subsequent proliferation and elongation of cells from behind the tip cell results in vessel stabilization and lumen formation, followed by lumen consolidation through fusion of the neovessel with the pre-existing vascular network (reviewed in Carmeliet and Jain, 2011). NMII coordinates these stages of angiogenesis, and thus exhibits complex spatial and temporal regulation depending on the process. For instance, localized NMII inhibition *in vitro* promotes angiogenic sprouting (Fischer et al., 2009) and initial vascular branching (Elliott et al., 2015) in 3D angiogenic models by decreasing the tension on actin stress fibers, which disrupts VE-cadherin-mediated intercellular bridges and weakens cell–cell adhesions (Hoelzle and Svitkina, 2012; reviewed in Yonemura, 2011). Simultaneously, at the tip cell, NMIIA and NMIIB activity are necessary for endothelial cell migration (Kolega, 2006). For the formation of the vascular lumen, NMII generates forces that stabilize VE-cadherin at cell–cell contacts (Fig. 4B), thus blocking further angiogenic sprouting (Abraham et al., 2009; Strilić et al., 2009). Interestingly, Nogo-A, a protein expressed by oligodendrocytes and neurons in the CNS, inhibits endothelial cell migration via a mechanism that involves RhoA–ROCK–NMII activation (Wälchli et al., 2013). This negative modulation of angiogenesis in the CNS compromises the repair of brain injuries. As a result of the complex regulation of NMII at diverse stages of blood vessel development, the modulation of myosin activity could be a powerful, yet still unexplored, tool for both the repression and induction of angiogenesis in disorders such as cancer, stroke and diabetic chronic wounds.

## NMII in other diseases

### MYH9-related diseases: NMII functions in development

Although this Review focuses primarily on emerging roles of NMII in genetically complex disorders, autosomal dominant mutations in NMHCIIA (Box 2) have provided fundamental insights into how altered NMII activity contributes to disease. These autosomal dominant NMIIA mutations result in various *MYH9-*related disorders (MYH9-RD), including May-Hegglin anomaly, Epstein syndrome, Fechtner syndrome and Sebastian syndrome; the hallmark features of which are fewer, but giant, platelets (referred to clinically as macrothrombocytopenia) and leukocyte inclusions. Individuals affected by a *MYH9-*related disorder often present with deafness, cataracts and nephropathy (Balduini et al., 2011). In the process of platelet formation, megakaryocytes extend pro-platelet projections into the bloodstream, where fluid shear stress results in their release and fragmentation into platelets (reviewed in Thon and Italiano, 2012). MYH9-RD megakaryocytes from affected humans and mouse models exhibit enlarged pro-platelet buds (Eckly et al., 2009; Pecci et al., 2009). Recent *in vitro* evidence demonstrates that fluid sheer stress locally activates cortical NMIIA, resulting in fragmentation of pro-platelets into platelets (Spinler et al., 2015). Decreased platelet number in MYH9-RD could also result from reduced collagen adhesion and premature platelet release outside the bloodstream (Balduini et al., 2011; Eckly et al., 2009), highlighting the need for *in vivo* disease models that capture the role of NMII in diverse events leading to disease pathology. The following section addresses how NMII contributes to body and tissue patterning, and highlights isoform-specific roles for NMII that help to explain tissue-specific defects in MYH9-RD (Kim et al., 2005).

### NMII in development and developmental disease

During development, NMII activity contributes to morphogenesis and tissue patterning. A recent study of individuals with congenital heart defects arising from incorrect left–right body patterning revealed that disease-associated CNVs commonly include an isoform of ROCK, ROCK2 (Fakhro et al., 2011). Although CNVs do not necessarily contribute to disease pathology, animal models provide further evidence for a role of ROCK-mediated myosin contractility in body axis asymmetry, and demonstrate that ROCK negatively regulates TGFβ signaling through receptor internalization and degradation (Wang et al., 2011, 2012b; Zhang et al., 2009). This is of particular interest because TGFβ serves as a critical regulator of other NMII-dependent processes discussed in this Review, including the epithelial-to-mesenchymal transition that underlies cancer metastasis (Xu et al., 2009) as well as stem cell renewal and differentiation (Watabe and Miyazono, 2009).

At the tissue level, NMII functions downstream of non-canonical Wnt planar cell polarity (PCP) pathways to regulate polarity. In the auditory epithelium, PCP pathways polarize NMII-driven contractile forces to pattern hair cells, which regulate sound perception (Lee et al., 2012; Yamamoto et al., 2009), providing a possible mechanism for the deafness associated with MYH9-RD. Similarly, in cardiac development, NMII activity functions downstream of PCP signaling in the polarized migration of the myocardium during the development of the heart's outflow tract, a process that relies primarily on NMIIB and possibly on NMIIC (Ma and Adelstein, 2012; Marigo et al., 2004; Phillips et al., 2005). However, NMIIA directs convergence and extension tissue movements during the development of the kidney tubules (Lienkamp et al., 2012), accounting for nephropathy in some MYH9-RD cases, characterized by altered NMIIA localization in renal structures and reduced NMIIA expression in podocytes (Ghiggeri et al., 2003). Altered NMII expression has been reported for several glomerular diseases (Hiroi et al., 1996; Miura et al., 2013). In addition to regulating tissue patterning, PCP-mediated NMII activity might also contribute to cancer progression, with more aggressive ovarian cancers showing increased PCP signaling pathways corresponding with changes in actomyosin organization (Feske et al., 2009). Thus, disrupted NMII activity results in developmental defects that provide insights into how NMII might contribute to the pathology of more genetically complex diseases, such as cancer.

## NMII in stem cell therapies

**Fig. 6.**
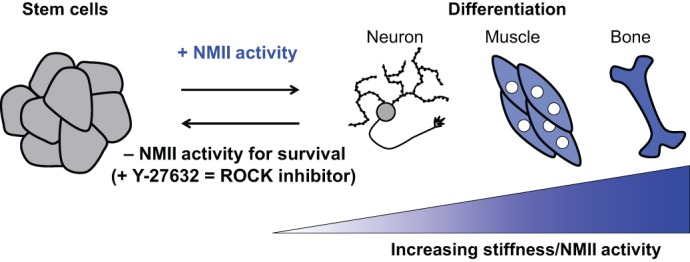
**NMII determines stem cell fate.** Pluripotent stem cells are cultured in the presence of the ROCK inhibitor Y-27632 to prevent apoptosis (Chen et al., 2010; Watanabe et al., 2007). NMII activity directs the differentiation of stem cells to specific tissue lineages: decreased NMII activity leads to neuronal fates, whereas increased NMII activity promotes the formation of stiffer tissues, such as muscle and bone (Engler et al., 2006; Seo et al., 2014; Wang et al., 2013b).

Given the increasing use of stem cells in research, it is imperative to understand the mechanisms that underlie the self-renewal of pluripotent stem cells and their directed differentiation to specific tissues in order to advance stem cell therapies. Recent findings demonstrate that NMII acts as an important regulator of both stem cell self-renewal and committed differentiation toward a specific cell type (Engler et al., 2006; Watanabe et al., 2007; Zhang and Kilian, 2013). For example, NMII activity in response to substrate stiffness directs the differentiation of mesenchymal stem cells to muscle and bone on stiffer substrates and to neurons on softer substrates (Engler et al., 2006). This suggests that altering the physiological stiffness of a stem cell's substrate and/or mimicking the activity of NMII to match that of a particular tissue might be sufficient to direct stem cells towards a particular fate (Fig. 6). In addition to discussing the mechanisms by which NMII differentially regulates stem cell self-renewal versus differentiation, we also discuss potential clinical applications of NMII inhibitors (Chen et al., 2014).

### NMII: a driver of differentiation

NMII regulates cell identity by several mechanisms, including signal mechanotransduction, transcriptional changes, ion-channel activation, and the regulation of cell–cell and cell–matrix interactions. When cells adhere to their ECM, external forces are transmitted through adhesion junctions to the inside of the cell, where NMII-dependent contractile forces balance them. Through mechanotransduction, these NMII-dependent contractile forces drive changes in signaling by regulating the composition of adhesion complexes. They also drive changes at the transcriptional level through mechanosensitive transcriptional coactivators, such as Yes-associated protein (YAP) (Dupont et al., 2011).

In stem cells, the nuclear translocation of YAP acts as a transcriptional regulator of self-renewal or differentiation into specific cell types. In multipotent mesenchymal stem cells, integrin-β1 activation results in RhoA–ROCK-mediated myosin activation and in the nuclear translocation of YAP, favoring differentiation to bone rather than to adipose or cartilage (Tang et al., 2013). Intriguingly, mesenchymal stem cells retain mechanical memory through YAP nuclear localization, such that culturing these cells on stiff substrates prior to their differentiation on soft substrates promotes osteogenesis rather than adipogenesis (Yang et al., 2014). However, inhibiting YAP nuclear localization by culturing cells on soft matrices promotes the robust differentiation of stem cells into post-mitotic neurons, even in the absence of neurogenic factors (Musah et al., 2014). This suggests that physical stiffness of the substrate is sufficient to drive the biochemical signaling events necessary for cells to differentiate into specific tissues (Engler et al., 2006; Musah et al., 2014). However, NMII-generated traction forces promote cortical neuron differentiation by activating the stretch-activated ion channel Piezo1, resulting in Ca^2+^ influx and YAP nuclear localization (Pathak et al., 2014). Thus, the specific function of YAP downstream of NMII activity likely results from cell-type-dependent mechanisms, such as specific transcription-factor interactions. This is illustrated in the intestinal epithelium, where YAP interaction with TEA domain (TEAD) transcription factors promotes stem cell self-renewal, whereas interaction with the transcription factor Kruppel-like factor 4 (Klf4) promotes differentiation into goblet cells (Imajo et al., 2014).

In addition to directing stem cell fate through transcriptional regulation, NMII also negatively regulates stem cell survival through apoptosis. RhoA–ROCK-mediated NMII activity contributes to apoptosis through nuclear fragmentation, membrane blebbing and the subsequent release of damaging proteins (Croft et al., 2005; Mills, 1998; Wickman et al., 2013). Because NMII-driven apoptosis limits stem cell viability, stem cell culture benefits from the use of either ROCK inhibitors, such as Y-267632, or NMII inhibitors, such as blebbistatin (Chen et al., 2010; Walker et al., 2010; Watanabe et al., 2007) (Fig. 6).

### Clinical applications of NMII inhibitors in stem cell therapies

Stem cell therapies hold promise for the treatment of numerous disorders, including cardiovascular disorders, spinal cord injuries and neurodegenerative diseases. However, several considerations limit the usefulness of stem cells in clinical settings, including the production of stem cells in sufficient quantities, the development of xeno-free culture systems, and their directed differentiation into the desired cell type. In each case, the manipulation of NMII activity is informing the development of technologies that advance research into stem cell therapies. For example, inhibition of ROCK-mediated NMII contractility allows for stem cell expansion on microcarriers in suspension, greatly enhancing stem cell production over traditional 2D culture methods (Chen et al., 2014). ROCK inhibition also allows for stem cell growth under xeno-free culture conditions (Harb et al., 2008). Finally, ROCK inhibition promotes neural differentiation of placental-derived multipotent cells to generate neural progenitor cells for the treatment of neurodegenerative disorders (Wang et al., 2013b). Alternatively, increased NMII activation, via fabricated micropits, promotes osteogenic differentiation of mesenchymal cells (Seo et al., 2014). Likewise, reducing substrate stiffness or inhibiting ROCK-mediated NMII activation promotes neural induction and the subsequent differentiation of neural stem cells to specific neuronal populations, such as motor neurons (Sun et al., 2014). Thus, in addition to pharmacological inhibition, fine-tuning NMII activity through engineered scaffolds of a particular stiffness or substrate holds promise for both stem cell expansion and their directed differentiation (Musah et al., 2014; reviewed in Murphy et al., 2014).

## Perspectives on NMII in disease

### Emerging regulators of NMII function as therapeutic targets

In this Review, we have sought to highlight the emerging roles for NMII in genetically complex disorders, such as cancer and neuronal diseases. In so doing, we have revealed that these diverse pathologies result from similar NMII-driven processes, including mechanical forces that drive dynamic cell movements and mechanotransduction that results in signaling changes that alter cell behavior. For example, NMII-mediated contractile forces drive both the cell migration that causes cancer metastasis and the synaptic plasticity that underlies cognitive function and that contributes to neurodevelopmental disorders (see Box 1). Similarly, biochemical signaling pathways downstream of NMII are shared across diverse disorders. In particular, recent evidence demonstrates that the mechanosensitive transcription factors YAP and transcriptional coactivator with PDZ-binding motif (TAZ; also known as WWTR1), which regulate both cancer cell progression and stem cell differentiation, also contribute to body axis patterning (Porazinski et al., 2015) and angiogenesis (Choi et al., 2015). Thus, understanding how altered NMII signaling contributes to a specific disease might provide insights into the mechanisms of other NMII-related disorders and ultimately help to identify novel therapeutic targets for the successful treatment of these disorders.

In an attempt to identify novel therapeutic targets for the treatment of NMII-related disorders, we have detailed common upstream regulatory mechanisms that govern NMII activity. These include diverse genetic mutations, as well as miRNAs, that converge either directly or indirectly on NMII regulation. Already, the miRNAs that regulate NMII activity in cancer and endothelial cell function (Table 3) have been implicated in stem cell pluripotency and differentiation (Mathieu and Ruohola-Baker, 2013), and are poised to provide further insights into the roles of NMII in these processes. Thus, miRNAs that regulate NMII function could serve as additional targets for directed differentiation to desired therapeutic cell types. Furthermore, the pharmacological inhibition of shared myosin kinases (Fig. 1) shows promise for therapeutic intervention in numerous disorders, while creating scaffolds of a particular stiffness provides an alternate strategy for manipulating NMII activity. Furthering our understanding of the particular molecular mechanisms that govern the precise spatial and temporal activity of NMII in normal and disease states should also provide increasing specificity for these therapeutic strategies.
